# Neonatal Intensive Care Unit Outbreak of *Ralstonia pickettii* Bacteremia Associated with Contaminated Sterile Distilled Water: Clinical, Environmental, and Molecular Epidemiological Investigation

**DOI:** 10.3390/pathogens15090953

**Published:** 2026-09-08

**Authors:** Saime Sundus Uygun, Hatice Turk Dagi, Gulsum Alkan, Onur Ural, Baris Otlu, Evrim Kilicli, Osman Selcuk Duysak, Hanifi Soylu, Murat Konak

**Affiliations:** 1 Division of Neonatology, Department of Pediatrics, Faculty of Medicine, Selcuk University, Konya 42130, Turkey; 2 Department of Medical Microbiology, Faculty of Medicine, Selcuk University, Konya 42130, Turkey; 3 Division of Pediatric Infectious Diseases, Department of Pediatrics, Faculty of Medicine, Selcuk University, Konya 42130, Turkey; 4 Department of Infectious Diseases, Faculty of Medicine, Selcuk University, Konya 42130, Turkey; 5 Department of Medical Microbiology, Faculty of Medicine, Inonu University, Malatya 44280, Turkey; 6 Division of Neonatology, Department of Pediatrics, University of Manitoba & Shared Health, Winnipeg, MB R3C 0C4, Canada

**Keywords:** environmental contamination, molecular epidemiology, neonatal intensive care unit, outbreak, *Ralstonia pickettii*

## Abstract

*Ralstonia pickettii* is an opportunistic Gram-negative bacterium associated with healthcare-associated infections, particularly in premature infants and other immunocompromised or critically ill patients. This study aimed to investigate a neonatal intensive care unit outbreak of *Ralstonia pickettii* bacteremia by describing the clinical characteristics of affected infants, identifying the environmental source, and evaluating the genetic relatedness between clinical and environmental isolates. Hospital records from January 2023 through November 2025 were reviewed; the nine included cases occurred between February 2024 and October 2025. Demographic, clinical, and laboratory data were reviewed. Environmental sampling was performed to identify the source of contamination. Genetic relatedness between clinical and environmental isolates was evaluated using arbitrarily primed polymerase chain reaction (AP-PCR), and the molecular findings were interpreted together with microbiological and epidemiological data. This study was initially designed as a retrospective clinical review and was subsequently expanded to include an outbreak investigation after three temporally clustered cases were identified in October 2025. The outbreak involved nine neonates with *Ralstonia pickettii* bacteremia. Environmental investigation identified *Ralstonia pickettii* in both opened and unopened sterile distilled water samples. Clinical and environmental isolates demonstrated highly similar AP-PCR banding patterns, supporting genetic relatedness when interpreted together with microbiological and epidemiological findings. Following removal of the contaminated source and implementation of infection control measures, no additional *R. pickettii* bacteremia cases were identified. Overall mortality was 44.4%, whereas only one death (11.1%) was considered attributable to *Ralstonia pickettii* bacteremia. *Ralstonia pickettii* can cause healthcare-associated outbreaks in neonatal intensive care units. Integration of microbiological, environmental, epidemiological, and molecular findings may facilitate timely outbreak source identification and implementation of effective infection control measures.

## 1. Introduction

Ralstonia species are non-fermentative Gram-negative bacilli belonging to the *Burkholderiaceae* family and are widely distributed in environmental sources. Although generally considered low-virulence organisms, they have emerged as important opportunistic pathogens causing healthcare-associated infections, particularly in immunocompromised patients and individuals exposed to invasive procedures [1]. Among the clinically relevant species, *Ralstonia pickettii* is the most frequently implicated in healthcare-associated outbreaks, although *R. insidiosa* and *R. mannitolilytica* have also been reported [2]. Their ability to survive for prolonged periods in water systems, distilled water, intravenous solutions, and disinfectants, together with their capacity to form biofilms on medical devices and artificial surfaces, enables persistence in hospital environments and facilitates environmental contamination [3,4].

These characteristics have been associated with contamination-related outbreaks in a variety of healthcare settings, including intensive care units, hemodialysis centers, and neonatal intensive care units (NICUs) [5,6]. In recent years, outbreaks linked to contaminated sterile solutions, intravenous fluids, and water-associated medical devices have been increasingly reported, highlighting the role of *Ralstonia* species as emerging healthcare-associated pathogens [7].

Newborns, particularly preterm infants, are highly susceptible to infections caused by opportunistic pathogens such as *Ralstonia* species because of their immature immune systems, prolonged hospital stays, and exposure to invasive procedures, including central venous catheterization, intubation, and total parenteral nutrition [8]. Accurate species identification remains challenging because conventional phenotypic methods may identify *Ralstonia* isolates as other non-fermentative Gram-negative bacilli. Consequently, the true incidence of *Ralstonia* infections may be underestimated [9]. In addition, limited antimicrobial susceptibility breakpoints and variable resistance patterns, particularly in neonatal patients, complicate treatment decisions [10].

This study aimed to investigate an outbreak of *R. pickettii* bacteremia in a neonatal intensive care unit by describing the clinical characteristics of affected infants, identifying the environmental source, and evaluating the genetic relatedness between clinical and environmental isolates using molecular epidemiological methods.

## 2. Materials and Methods

### 2.1. Study Design and Setting

This study was initially designed as a retrospective review of the demographic and clinical characteristics of neonates with *Ralstonia pickettii* bacteremia treated in the neonatal intensive care unit (NICU) of Selçuk University Faculty of Medicine Hospital, Konya, Turkey. During the study period, three additional cases were identified within a short interval in October 2025, raising concern for a healthcare-associated outbreak and expanding the scope of the study to include an outbreak investigation. Accordingly, the retrospective clinical review was supplemented by environmental sampling, infection control surveillance, and molecular epidemiological analysis to identify the potential source of contamination and assess the relatedness between clinical and environmental isolates.

### 2.2. Case Definition and Inclusion Criteria

Neonates with blood culture-confirmed *R. pickettii* bacteremia accompanied by clinical and laboratory findings consistent with infection were eligible for inclusion in this outbreak investigation.

The distinction between true bacteremia and possible contamination or pseudobacteremia was assessed based on both clinical and laboratory findings. Cases with a single positive blood culture in the absence of clinical signs of infection (apnea, bradycardia, feeding intolerance, increased need for respiratory support, or temperature instability) and without laboratory evidence of infection (elevated C-reactive protein [CRP], abnormal white blood cell count, or thrombocytopenia) were considered contamination and excluded.

Clinical sepsis was defined as the presence of one or more clinical manifestations together with at least one laboratory abnormality consistent with infection. Cases with polymicrobial bloodstream infections were excluded.

### 2.3. Microbiological Methods

Blood cultures were obtained under aseptic conditions from peripheral veins and/or existing intravascular catheters in neonates with suspected sepsis and incubated in the BACT/ALERT^®^ 3D automated blood culture system (bioMérieux, Marcy-l’Étoile, France). Bottles yielding positive signals were subjected to Gram staining, and samples were subcultured onto 5% sheep blood agar and eosin methylene blue (EMB) agar plates. The inoculated plates were incubated at 37 °C for 18–24 h and examined for bacterial growth.

Representative colonies were identified by matrix-assisted laser desorption/ionization time-of-flight mass spectrometry (MALDI-TOF MS) using the VITEK MS system (bioMérieux, France), a method previously used for the identification of clinically relevant *Ralstonia* species [11]. Spectra were analyzed using the VITEK MS reference database, and only identifications meeting the manufacturer-recommended confidence criteria were accepted.

All clinical and environmental isolates identified as *R. pickettii* were reviewed by experienced clinical microbiologists before inclusion in the molecular analysis. Only isolates yielding consistent species-level MALDI-TOF MS identification and supported by the available microbiological and epidemiological data were included.

### 2.4. Environmental Investigation

Following recognition of a temporal cluster of *R. pickettii* bacteremia cases, an environmental investigation was initiated in collaboration with the hospital infection control committee. Environmental sampling sites were selected based on their potential role in water-associated transmission within the neonatal intensive care unit (NICU).

The initial environmental investigation was conducted on 4 November 2025 and included sampling from incubator humidifiers, ventilator humidification units, oxygen flowmeters, and used fluid preparations. To further investigate the potential contamination source, a second and more comprehensive investigation was carried out on 18 November 2025, during which water systems, humidifier units, and sterile distilled water from both opened and unopened containers were re-evaluated.

To identify the contamination source, opened and unopened sterile distilled water samples were processed under aseptic conditions in a biosafety cabinet. Following centrifugation, the sediment was inoculated onto 5% sheep blood agar and eosin methylene blue (EMB) agar plates using standard microbiological procedures. The recovered isolates were subsequently confirmed by the microbiology laboratories of two independent tertiary-care hospitals: one university hospital and one tertiary-care city hospital.

The sterile distilled water was a commercially manufactured product procured by the hospital through the institutional tender process and was not prepared or sterilized within the hospital. Following the identification of *R. pickettii* contamination in the product, the findings were formally documented, and the relevant official procedures were initiated. The hospital administration and relevant local health authorities, including the Provincial Directorate of Health, were notified.

### 2.5. Molecular Analysis

Genetic relatedness between clinical and environmental *R. pickettii* isolates recovered during the outbreak was evaluated using arbitrarily primed polymerase chain reaction (AP-PCR). AP-PCR was performed according to a previously published protocol [11], using the M13 primer (5′-GAGGGTGGCGGTTCT-3′) for amplification. PCR reactions consisted of an initial denaturation step at 94 °C for 5 min, followed by 35 cycles of denaturation at 94 °C for 1 min, annealing at 36 °C for 1 min, and extension at 72 °C for 2 min, with a final extension step at 72 °C for 10 min. Amplified products were separated by electrophoresis on 1.5% agarose gels and visualized under ultraviolet illumination.

Banding patterns were visually compared to assess genetic relatedness among isolates. AP-PCR was used for comparative fingerprinting of the previously identified isolates and was not intended for species identification. Isolates exhibiting indistinguishable or highly similar AP-PCR banding patterns were interpreted as genetically closely related within the context of the outbreak investigation.

Due to technical and financial constraints, AP-PCR typing was performed on a selected subset comprising five clinical isolates obtained from five different neonates and five environmental isolates, rather than on all available clinical isolates.

### 2.6. Data Collection

Demographic, clinical, and laboratory data were retrospectively collected from hospital electronic medical records and patient files. Demographic variables included gestational age, birth weight, sex, mode of delivery, and underlying comorbidities. Clinical variables and potential risk factors included the presence of a central venous catheter, urinary catheter, mechanical ventilation, nasogastric/orogastric tube use, total parenteral nutrition, and previous broad-spectrum antibiotic exposure.

Laboratory variables included C-reactive protein (CRP), white blood cell (WBC) count, absolute neutrophil count (ANC), platelet count, blood culture results, and meropenem minimum inhibitory concentration (MIC) values. Clinical outcome measures included time to blood culture negativity, length of hospital stay, overall mortality, and outbreak-related mortality.

### 2.7. Ethical Approval

The study was approved by the Local Ethics Committee of the Faculty of Medicine, Selçuk University (Approval No. 2025/440; 29 July 2025) and was conducted in accordance with the principles of the Declaration of Helsinki. Written informed consent for participation was obtained from the parents or legal guardians of all enrolled neonates.

### 2.8. Statistical Analysis

Data were analyzed using IBM SPSS Statistics for Windows, version 21.0 (IBM Corp., Armonk, NY, USA). Given the descriptive nature of this outbreak investigation and the limited number of cases, only descriptive statistical analyses were performed. Continuous variables are presented as mean ± standard deviation (SD) or median (range), as appropriate, whereas categorical variables are presented as frequencies and percentages.

### 2.9. Use of Generative Artificial Intelligence

A generative artificial intelligence-assisted tool (OpenAI, San Francisco, CA, USA; https://openai.com/codex/; accessed on 31 August 2026) was used during manuscript revision to assist with English-language editing, improve the clarity and organization of selected passages, and support the visual organization of the epidemiological timeline. The tool was not used to generate or modify the underlying clinical, microbiological, environmental, or molecular data and was not used to perform statistical analyses. All AI-assisted outputs were critically reviewed, verified, and revised by the authors, who take full responsibility for the accuracy and integrity of the manuscript.

## 3. Results

### 3.1. Clinical Characteristics of the Affected Neonates

A total of nine neonates with culture-confirmed *R. pickettii* bacteremia were included in the outbreak investigation. The mean gestational age was 32.9 ± 3.5 weeks (range, 25–38 weeks), and the mean birth weight was 1723.3 ± 656.3 g (range, 700–2700 g). Eight infants (88.9%) were born preterm, and five (55.6%) were female. Delivery was by cesarean section in seven infants (77.8%). Underlying comorbidities were present in five infants (55.6%). The most common potential risk factors were nasogastric/orogastric tube use (66.7%), central venous catheterization (55.6%), and mechanical ventilation (55.6%). Demographic, perinatal, and baseline clinical characteristics are summarized in Table 1.

### 3.2. Laboratory Findings and Clinical Outcomes

Laboratory findings and clinical outcomes are summarized in Table 2. The median C-reactive protein (CRP) level was 57 mg/L (range, 13–130 mg/L). The mean white blood cell count was 11,166.7 ± 8361.6/µL, the mean absolute neutrophil count was 7067.8 ± 6821.3/µL, and the mean platelet count was 217,222 ± 168,156/µL. A comprehensive antimicrobial susceptibility profile was not available for the *R. pickettii* isolates; therefore, meropenem MIC values were reported for all nine bloodstream isolates. The individual meropenem MIC values were 32, 0.25, 1, 0.5, 4, 4, 8, 0.75, and 4 mg/L, respectively, with a median MIC of 4 mg/L (range, 0.25–32 mg/L). Because standardized interpretive breakpoints for *R. pickettii* are limited, categorical susceptibility classifications were not assigned. Median time to blood culture negativity was 9.5 days (range, 3–32 days). One patient died before microbiological clearance could be documented and was therefore excluded from this analysis. The median length of hospital stay was 50 days (range, 8–225 days). Overall mortality was 44.4% (4/9), whereas one death (11.1%) was considered attributable to *R. pickettii* bacteremia (Table 2).

### 3.3. Environmental Investigation and Molecular Analysis

The temporal clustering of three *R. pickettii* bacteremia cases, identified from blood cultures collected on 2, 13, and 28 October 2025, prompted a formal outbreak investigation, including environmental sampling. *R. pickettii* was recovered from incubator humidifiers, oxygen flow meters, ventilator humidification units, and both opened and unopened bottles of sterile distilled water.

Following the isolation of *R. pickettii* from opened sterile distilled water bottles, three additional unopened bottles from the same product batch were aseptically opened and cultured in the hospital microbiology laboratory. *R. pickettii* was recovered from all three bottles. To independently verify these findings, unopened bottles were subsequently examined in the microbiology laboratories of two independent tertiary-care hospitals—one university hospital and one tertiary-care city hospital—where *R. pickettii* was again recovered. These findings strongly suggested that contamination occurred before use of the product rather than during handling within the NICU.

During the initial environmental investigation, several additional microorganisms, including *Pseudomonas aeruginosa*, *Klebsiella pneumoniae*, *Serratia marcescens*, *Sphingomonas paucimobilis*, and *Stenotrophomonas maltophilia*, were isolated from other environmental samples. However, *R. pickettii* was detected exclusively in water-containing environments associated with sterile distilled water use.

**Figure 1:**
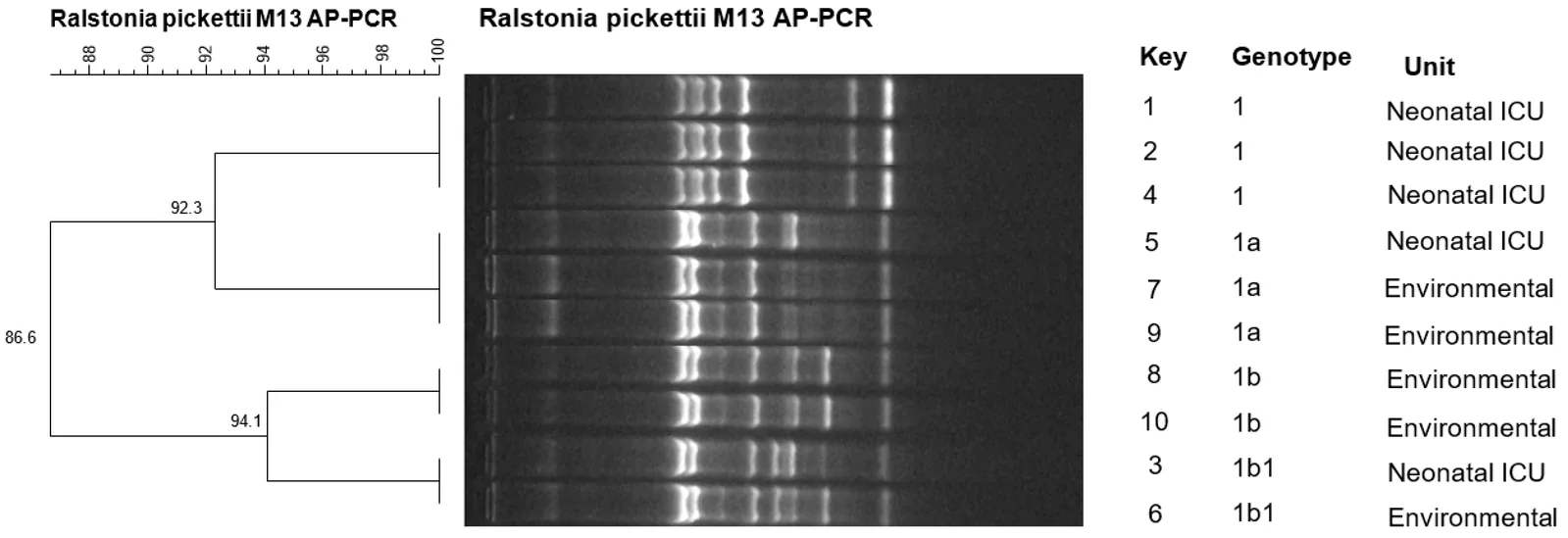
AP-PCR fingerprinting profiles of representative clinical and environmental *R. pickettii* isolates. Lanes 1–10, representative clinical and environmental isolates. The AP-PCR profiles demonstrate genetic relatedness among the isolates; the bands do not represent species-specific diagnostic markers.

Clinical and environmental isolates demonstrated highly similar AP-PCR banding patterns. When interpreted together with the microbiological, temporal, and epidemiological findings, these molecular results supported genetic relatedness between the clinical and environmental isolates (Figure 1).

Review of hospital microbiology records identified no additional *Ralstonia* bloodstream infections in other hospital units during the outbreak investigation period, indicating that the outbreak was confined to the neonatal intensive care unit.

### 3.4. Outbreak Control and Follow-Up

**Figure 2:**
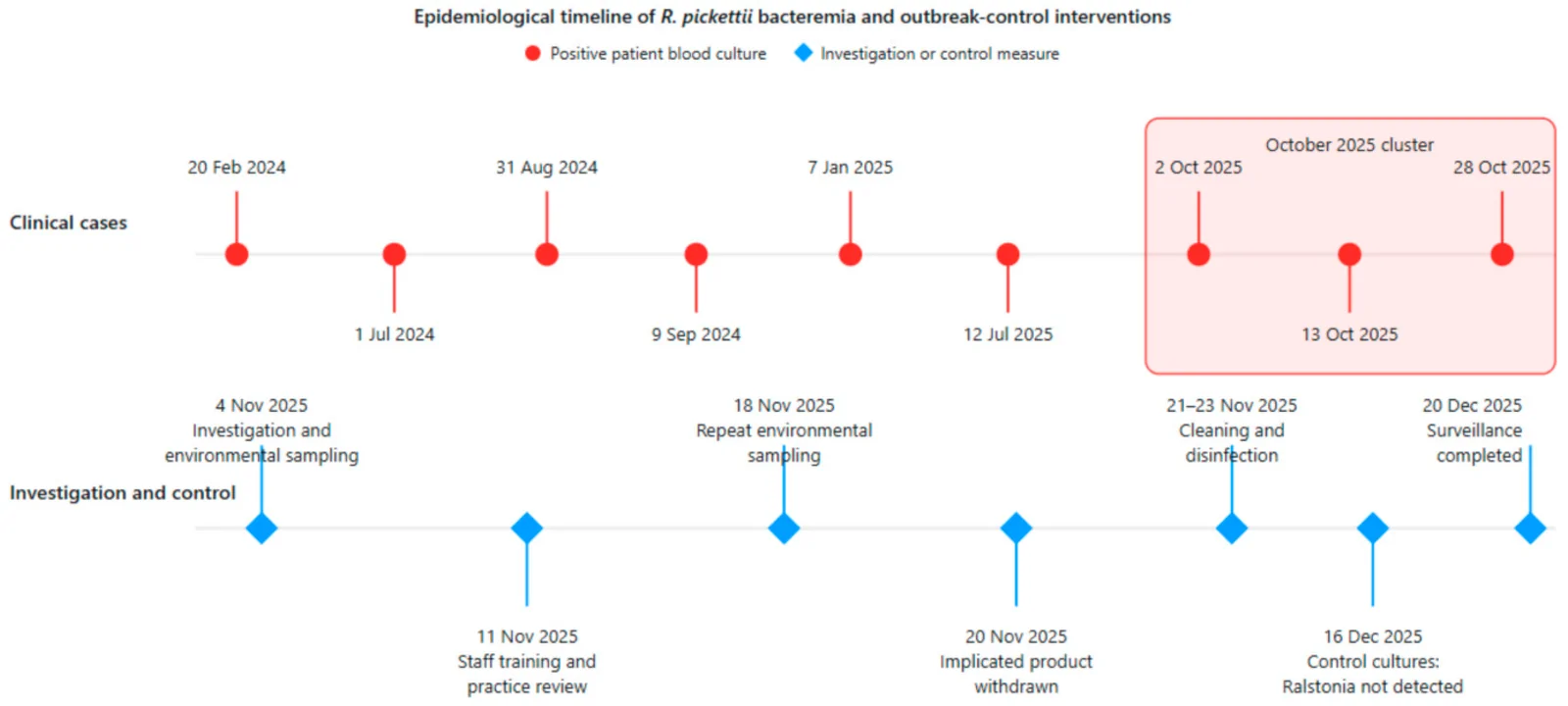
Epidemiological timeline of *Ralstonia pickettii* bacteremia cases and principal outbreak-control interventions. Red circles indicate positive patient blood cultures, and blue diamonds indicate investigation or outbreak-control measures. The light-red shaded box indicates the temporal cluster of the final three R. pickettii bacteremia cases identified in October 2025, which prompted the formal outbreak investigation.

The temporal distribution of *R. pickettii* bacteremia cases and the principal outbreak-control interventions is presented in Figure 2. Six cases were identified at intervals between February 2024 and July 2025. Three additional cases were subsequently identified from blood cultures collected on 2, 13, and 28 October 2025, indicating temporal clustering and prompting a formal infection control investigation.

Environmental sampling was initiated on 4 November 2025. *R. pickettii* was recovered from opened bottles of sterile distilled water and water-containing equipment used in the NICU. Repeat environmental sampling subsequently yielded *R. pickettii* from an unopened bottle of the implicated sterile distilled water product. The product was withdrawn from clinical use on 20 November 2025. *R. pickettii* was also recovered from additional unopened bottles from the same product batch, and the finding was independently confirmed by the microbiology laboratories of two separate hospitals.

Healthcare personnel received additional training in hand hygiene, aseptic technique, and device-handling practices on 11 November 2025. Contact precautions were implemented for all patients, and patient cohorting was performed when necessary. Between 21 and 23 November 2025, the NICU underwent cleaning and disinfection. Surfaces, medical devices, and patient-care areas were reprocessed in accordance with institutional infection control protocols, all fluid sources were replaced, and the sterile distilled water supply chain was reviewed.

Active surveillance continued until 20 December 2025. Blood culture results were reviewed daily, and repeat environmental sampling was performed to assess elimination of the contamination source. *Ralstonia* spp. was not detected in 25 control environmental samples collected on 16 December 2025, and no additional cases of *R. pickettii* bacteremia were identified during the post-intervention surveillance period.

Nine cases were identified between February 2024 and October 2025. The timeline shows the temporal clustering of the final three cases and the subsequent investigation, product withdrawal, cleaning and disinfection, control environmental sampling, and active surveillance.

## 4. Discussion

In this study, we investigated a healthcare-associated outbreak of *R. pickettii* bacteremia in a neonatal intensive care unit through an integrated clinical, environmental, and molecular epidemiological approach. *R. pickettii* was recovered from both clinical bloodstream isolates and multiple environmental sources, including opened and unopened bottles of sterile distilled water and associated humidification systems. Taken together, the environmental and molecular findings, interpreted in the context of the microbiological, epidemiological, and temporal data, supported contaminated sterile distilled water as the most likely source of the outbreak.

The affected cohort consisted predominantly of preterm infants with multiple invasive devices, consistent with the well-recognized risk profile for *R. pickettii* healthcare-associated infections. Prematurity, together with the frequent use of central venous catheters, mechanical ventilation, and nasogastric/orogastric tubes, likely increased susceptibility to bloodstream infection by facilitating exposure to opportunistic pathogens and compromising host defense mechanisms [12,13]. Our findings are consistent with those of Burzyńska et al., who described a neonatal intensive care unit outbreak in which mechanical ventilation and central venous catheterization were among the most common predisposing factors [12].

In the present outbreak, *R. pickettii* was recovered from both opened and unopened bottles of sterile distilled water as well as from humidification systems, strongly suggesting a water-associated environmental source. Following the initial isolation, additional unopened bottles from the same product batch were cultured under sterile laboratory conditions in our microbiology laboratory, and *R. pickettii* was recovered from all tested bottles. Independent confirmation by the microbiology laboratories of two separate tertiary-care hospitals—one university hospital and one tertiary-care city hospital—further supported these findings. Collectively, these results strongly suggested that contamination had occurred before clinical use of the product and made contamination during routine handling within the NICU highly unlikely. However, the precise stage at which contamination occurred, whether during manufacturing, packaging, or distribution, could not be determined within the scope of the present investigation. The findings were therefore reported to the appropriate public health authorities for further investigation of the implicated product. Similar environmental reservoirs have been reported in previous healthcare-associated outbreaks involving *Ralstonia* species. *R. pickettii* has been linked to contaminated intravenous fluids and medical equipment, whereas *R. mannitolilytica* and *R. insidiosa* have been associated with contaminated water systems, intravenous solutions, and arterial blood gas syringes [14,15,16,17]. Taken together, these observations emphasize the remarkable ability of *Ralstonia* species to survive in aqueous environments. They also highlight the importance of combining hospital-based environmental sampling with formal product trace-back, lot-level microbiological testing, and, where indicated, investigation of manufacturing, packaging, and distribution processes by the relevant authorities when clusters of bloodstream infections are recognized.

Identification of contaminated sterile distilled water through a comprehensive environmental investigation enabled prompt source control and implementation of targeted infection control measures. Following removal of the contaminated source and implementation of comprehensive infection control measures, *R. pickettii* was no longer recovered in subsequent environmental sampling, and no additional outbreak-related cases were identified during the surveillance period. These findings highlight the importance of early environmental investigation and timely source control for successful containment of healthcare-associated *Ralstonia pickettii* outbreaks [18].

AP-PCR analysis provided supportive molecular evidence of genetic relatedness between representative bloodstream and environmental *R. pickettii* isolates. However, given the limited discriminatory power of AP-PCR compared with sequence-based typing methods, the molecular findings were interpreted together with the microbiological, epidemiological, and temporal evidence rather than as definitive proof of clonality. Although higher-resolution methods such as whole-genome sequencing or multilocus sequence typing would provide a more robust assessment of clonal relationships, AP-PCR served as a practical tool to complement the epidemiological investigation in the present outbreak [19].

Overall mortality in our cohort was 44.4%, whereas only one death (11.1%) was considered directly attributable to *R. pickettii* bacteremia. The remaining deaths occurred after microbiological clearance of the infection and were considered more likely to be related to prematurity and severe underlying clinical conditions rather than persistent bacteremia. These findings emphasize that, although *R. pickettii* may cause serious healthcare-associated infections in vulnerable neonates, clinical outcomes are also substantially influenced by the patients’ baseline characteristics and comorbidities [20].

The fatal outcome observed in one neonate may be explained, at least in part, by the markedly increased vulnerability of this infant. This neonate was born at 25 weeks of gestation with a birth weight of 700 g, representing the most immature and lowest-birth-weight infant in the series. At the time of bacteremia, the infant was intubated and had a central venous catheter, was receiving total parenteral nutrition, and had a nasogastric tube. In addition, sepsis developed on postnatal day 5, representing the earliest onset of sepsis among the neonates in the series. Extreme prematurity, very low birth weight, immature immune function, and the need for invasive support may have collectively increased the infant’s vulnerability to severe infection and an unfavorable outcome. In contrast, the other neonates may have had greater physiological and immunological reserves and less severe underlying vulnerability at the time of infection. Nevertheless, given the retrospective nature and small sample size of this outbreak investigation, the fatal outcome cannot be attributed solely to *R. pickettii* bacteremia and should be considered multifactorial.

To the best of our knowledge, this study represents one of the few reported neonatal *R. pickettii* outbreaks in which a comprehensive environmental investigation was integrated with molecular epidemiological analysis of both clinical and environmental isolates. By combining clinical, microbiological, environmental, and molecular findings, our investigation was able to identify the most likely source of the outbreak and facilitate targeted infection control interventions. These findings reinforce the importance of early environmental investigation, systematic surveillance, and multidisciplinary collaboration in the management of healthcare-associated outbreaks in neonatal intensive care units.

This study has several limitations. First, its retrospective, single-center design and the small number of affected neonates limit the generalizability of the findings. Second, species identification was based on MALDI-TOF MS and was not confirmed by 16S rRNA gene sequencing or whole-genome sequencing. Although MALDI-TOF MS generally provides rapid and accurate identification of non-fermenting bacteria, discordant or unidentified results have been reported compared with 16S rRNA gene sequencing, particularly for uncommon species and depending on the coverage of the reference database [21]. Therefore, the possibility of species-level misidentification cannot be completely excluded. Third, AP-PCR was performed on representative available bloodstream and environmental isolates rather than on all outbreak isolates. Because AP-PCR has lower discriminatory power than higher-resolution sequence-based typing methods, the observed similarities in banding patterns should be interpreted as supportive evidence of genetic relatedness rather than definitive confirmation of clonality. Accordingly, contaminated sterile distilled water was considered the most likely outbreak source based on the combined microbiological, environmental, temporal, and epidemiological evidence rather than on the molecular findings alone. Finally, attribution of mortality to the outbreak organism was based on clinical assessment and the temporal relationship between infection, microbiological clearance, and death and may therefore be subject to classification uncertainty. An additional limitation is that blood cultures were obtained from peripheral veins and/or existing intravascular catheters. Although specimens were collected under aseptic conditions, blood sampling through intravascular catheters may theoretically introduce a risk of contamination. However, the repeated recovery of *R. pickettii* from multiple environmental sources, including unopened sterile distilled water bottles, together with the supportive molecular and epidemiological findings, makes contamination during blood culture collection alone an unlikely explanation for the outbreak. Nevertheless, this possibility cannot be completely excluded. In addition, a molecular size marker was not included in the original AP-PCR gel; therefore, individual band sizes could not be determined, limiting the standardization and reproducibility of the banding-pattern comparison. Nevertheless, the integration of clinical data, systematic environmental investigation, molecular typing, and post-intervention surveillance represents an important strength of the study.

## 5. Conclusions

This outbreak investigation identified contaminated sterile distilled water as the probable source of *R. pickettii* transmission in the NICU, supported by microbiological, epidemiological, and molecular findings. These findings emphasize the importance of prompt environmental investigation, source control, and careful surveillance of water-containing products and humidification equipment in neonatal intensive care settings.

## Figures and Tables

**Table 1:** Demographic, oerinatal, and baseline clinical characteristics of neonates with *R. pickettii* bacteremia (*n* = 9).

Characteristic	Value
Gestational age, weeks	32.9 ± 3.5 (25–38)
Birth weight, g	1723.3 ± 656.3 (700–2700)
Female sex	5 (55.6%)
Cesarean delivery	7 (77.8%)
Preterm birth (<37 weeks)	8 (88.9%)
Underlying comorbidities	5 (55.6%)
Central venous catheter	5 (55.6%)
Urinary catheter	1 (11.1%)
Mechanical ventilation	5 (55.6%)
Nasogastric/orogastric tube	6 (66.7%)
Total parenteral nutrition	3 (33.3%)
Previous broad-spectrum antibiotic exposure	4 (44.4%)
Age at sepsis onset, days	29 (5–198)

Data are presented as mean ± standard deviation (range), median (range), or *n* (%), as appropriate. Underlying comorbidities included cleft lip and palate with ventricular septal defect, schizencephaly, perinatal asphyxia with cerebral palsy, biliary atresia, isolated ventricular septal defect, and esophageal atresia.

**Table 2:** Laboratory Findings, Antimicrobial Treatment, and Clinical Outcomes of Neonates with *R. pickettii* Bacteremia (*n* = 9).

Characteristic	Value
C-reactive protein (CRP), mg/L	57 (13–130)
White blood cell count (/µL)	11,166.7 ± 8361.6 (2210–30,950)
Absolute neutrophil count (/µL)	7067.8 ± 6821.3 (750–23,190)
Platelet count (/µL)	217,222 ± 168,156 (54,000–629,000)
Meropenem MIC (mg/L)	4 (0.25–32)
Time to blood culture negativity, days †	9.5 (3–32)
Length of hospital stay, days ‡	50 (8–225)
Overall mortality	4 (44.4%)
Outbreak-related mortality	1 (11.1%)

Data are presented as mean ± standard deviation (SD) (range), median (range), or *n* (%), as appropriate. † Calculated among patients with documented microbiological clearance. One patient died before microbiological clearance and before the final identification of the causative organism became available. Therefore, time to blood culture negativity could not be determined for this patient, although all available clinical, microbiological, and outcome data were included in the study. ‡ Calculated from admission until hospital discharge or death.

## Data Availability

The datasets generated and/or analyzed during the current study are not publicly available because of patient confidentiality but may be obtained from the corresponding author upon reasonable request and subject to applicable ethical and institutional approvals.
